# 

**Published:** 1851-10

**Authors:** 


### THE DENTAL REGISTER OF THE WEST.

JAMES TAYLOR M. D., D. D. S., EDITOR.

**PUBLISHED QUARTERLY;**

BY ORDER OF THE MISSISSIPPI VALLEY ASSOCIATION OF DENTAL SURGEONS.

CINCINNATI:
PRINTED BY JOHN D. THORPE,
NO. 74, WEST FOURTH STREET.
1851.

TERMS---$2,00 PER ANNUM, PAYABLE IN ADVANCE.

